# Optical Properties of Mn-Doped CuGa(In)S-ZnS Nanocrystals (NCs): Effects of Host NC and Mn Concentration

**DOI:** 10.3390/nano12060994

**Published:** 2022-03-17

**Authors:** Bryan Lee, Tristan Hegseth, Xiaoshan Zhu

**Affiliations:** 1 Department of Electrical and Biomedical Engineering, University of Nevada Reno, Reno, NV 89557, USA; bryanlee@nevada.unr.edu (B.L.); tristanhegseth@nevada.unr.edu (T.H.); 2 Biomedical Engineering Program, University of Nevada Reno, Reno, NV 89557, USA

**Keywords:** time-gated fluorescence measurement, Mn doping, nanocrystals

## Abstract

Time-gated fluorescence measurement (TGFM) using long-life fluorescence probes is a highly sensitive fluorescence-measurement technology due to the inherently high signal-to-background ratio. Although many probes for TGFM such as luminescent-metal-complex probes and lanthanide-doped nanoparticles are in development, they generally need sophisticated/expensive instruments for biosensing/imaging applications. Probes possessing high brightness, low-energy (visible light) excitation, and long lifetimes up to milliseconds of luminescence, are highly desired in order to simplify the optical and electronic design of time-gated instruments (e.g., adopting non-UV-grade optics or low-speed electronics), lower the instrument complexity and cost, and facilitate broader applications of TGFM. In this work, we developed Mn-doped CuGa(In)S-ZnS nanocrystals (NCs) using simple and standard synthetic steps to achieve all the desired optical features in order to investigate how the optical properties (fluorescence/absorption spectra, brightness, and lifetimes) of the Mn-doped NCs are affected by different host NCs and Mn concentrations in host NCs. With optimal synthetic conditions, a library of Mn-doped NCs was achieved that possessed high brightness (up to 47% quantum yield), low-energy excitation (by 405 nm visible light), and long lifetimes (up to 3.67 ms). Additionally, the time-domain fluorescence characteristics of optimal Mn-doped NCs were measured under pulsed 405 nm laser excitation and bandpass-filter-based emission collection. The measurement results indicate the feasibility of these optimal Mn-doped NCs in TGFM-based biosensing/imaging.

## 1. Introduction

Time-gated fluorescence measurement (TGFM) using long-life fluorescence probes is an important biosensing/imaging technology [1,2]. In TGFM, following pulsed excitation, the sample autofluorescence fades out in tens of nanoseconds, but the probes can continue emitting fluorescence up to milliseconds. By detecting the time-gated emission from the probes, the signal-to-background ratio can be significantly enhanced to ensure high-quality sensing/imaging. For the development of such probes, significant efforts have been focused on luminescent metal-complex probes (organic molecules coordinating a lanthanide or transition metal through electrostatic interaction) due to their unique spectral and temporal properties [3]. Lanthanide complexes have fluorescence lifetimes of microseconds to milliseconds, but they need UV light for excitation, which would complicate the optical design of the time-gated-instrument development, specifically in compact/portable instruments for point-of-care applications [3,4,5,6,7]. Transition-metal-complexes can be excited with visible light, but usually have lifetimes of a hundred or thousand nanoseconds. Such short lifetimes of transition-metal-complexes require high-speed electronics for signal recording and thus increase the instrument complexity and cost [3]. Besides luminescent metal-complex probes, lanthanide-doped polymer particles, with both silica nanoparticles and fluoride nanoparticles, with long fluorescence lifetimes are also in development [8,9,10,11]. Lanthanide-doped polymer particles with silica nanoparticles still need high energy for excitation as lanthanide complexes. For lanthanide-doped fluoride nanoparticles, although they can be excited by near-infrared light through up-conversion for bioapplications, they suffer from low brightness and need high-power excitation sources with complex and bulky optics and electronics [10,11]. It is preferred to have probes with high brightness, low-energy (visible light) excitation, and long lifetimes up to milliseconds. From a viewpoint of materials science and engineering, new material candidates in the nano scale need to be explored in order to achieve the desired features (high brightness, low-energy excitation, and long lifetimes up to milliseconds). With recent advances in nanocrystal research, Mn-doped nanocrystals (NCs) are promising for this purpose.

For Mn-doped NCs, once the exciton energy is transferred to the ^4^T_1_ state of the Mn dopants, the spin relaxation and slow inversion between the ^4^T_1_ and ^6^A_1_ states of Mn should cause an ideal yellow emission at ~585 nm with a long lifetime [12]. Mn elements have been doped into different types of NCs including binary NCs (e.g., CdSe or ZnSe NCs) or multinary NCs (e.g., I(II)-III-VI NCs) [12,13,14,15,16,17,18,19,20,21]. When Mn is doped into binary excitonic NCs, the excitonic fluorescence of host NCs will be completely quenched and only the Mn emission should emerge, because the transfer of the exciton energy to the Mn ^4^T_1_ state is much faster compared to the excitonic recombination of host NCs [13,14,15,16,17,18,19,20,21]. With respect to their optical properties, the absorption spectra of Mn-doped binary excitonic NCs are still determined by the bandgap of these host NCs, but the fluorescence brightness and lifetime of the doped NCs will be mainly affected by the Mn–Mn interaction in these host NCs [20,21]. Generally, Mn-doped binary NCs have average fluorescence lifetimes in the range of sub-milliseconds and need high energy for excitation, or they contain the toxic elements of Cd and/or Se against biosensing/imaging applications.

Among many multinary NCs, I(II)-III-VI NCs such as Cu-In-S/ZnS and Cu-Zn-In-S/ZnS NCs do not contain any Cd or Se elements and are particularly attractive to biomedical applications [22,23,24,25,26]. Different from binary excitonic NCs, they are donor–acceptor-pair-model-based materials. In these NCs, the donor states could be attributed to sulfur vacancies, silver/copper interstitials and gallium/indium atoms occupying substituted silver/copper sites, and the acceptor states could be from indium/gallium interstitials and silver/copper atoms occupying gallium/indium sites [22,23,24,25,26,27,28]. The fluorescence of I(II)-III-VI NCs should be mainly attributed to the electron transition from the donor states to acceptor states [22,23,24,25,26,27,28,29]. Moreover, the optical properties of I(II)-III-VI NCs can be adjusted by diverse compositional combinations among monovalent copper/silver and trivalent indium/gallium, as well as further monovalent-to-trivalent cation stoichiometric variation [30]. For Mn-doped I(II)-III-VI NCs, upon photoexcitation, the excitation energy of the host I(II)-III-VI NCs can be transferred to both Mn dopants and donor states, which could result in both Mn emission and donor–acceptor emission, or the dominant Mn emission. Additionally, the microenvironments (e.g., defects, electronic structures) in the I(II)-III-VI host NCs, which are determined by compositional combination and element stoichiometric variation in NCs, would significantly affect the lifetime of the Mn emission [12,31]. Considering all these factors, the optical properties (fluorescence/absorption spectra, brightness and lifetimes) of Mn-doped I(II)-III-VI NCs would be versatile but also complex. For instance, some works on Mn-doped I(II)-III-VI NCs have been reported, but on the basis of their own particular synthetic approaches, they presented significant changes in fluorescence lifetimes (hundreds of nanoseconds to milliseconds) and fluorescence wavelengths (yellow to red), presented complex steps in synthesis, or still needed high energy (UV light) for NC excitation due to the adoption of wide-bandgap NCs [32,33,34,35,36]. Some studies intended to synthesize NCs with double emission peaks from both Mn emission and donor–acceptor emission for photovoltaic applications (e.g., white LEDs) [37,38,39,40].

Specific to the applications in time-gated measurements, more efforts are still needed to investigate Mn-doped I(II)-III-VI NCs towards achieving the desired features (high brightness, low-energy excitation, and long lifetimes up to milliseconds) as time-gated probes. In this work, on the basis of a simple synthetic approach using standard steps, aiming to achieve Mn-doped I(II)-III-VI NCs with the desired optical features, our research objective was to systematically investigate how the optical properties (fluorescence/absorption spectra, brightness, and lifetimes) of Mn-doped NCs are affected by different host NCs and Mn concentrations.

## 2. Materials and Methods

### 2.1. Materials and Apparatus

Copper (I) iodide (CuI, 99%), manganese acetate (Mn(Ac)_2_, 99%), gallium (III) acetylacetonate (Ga(acac)_3_, 99%), zinc acetate (Zn(Ac)_2_, 99%), and indium (III) acetate (In(Ac)3, 99.99%) were from Alfa Aesar (Haverhill, MA, USA). Oleylamine (70%), 1-octadecene (ODE, 90%), 1-dodecanethiol (DDT, 98%), oleic acid (90%), and sulfur were purchased from Sigma Aldrich (St. Louis, MO, USA).

A RF-5301PC spectrophotometer (Shimadzu, Kyoto, Japan) was used for the measurement of the emission spectra of NCs. A UV-2450 spectrometer (Shimadzu, Kyoto, Japan) was adopted for the measurement of the UV-Vis absorption spectra. The excited-state decay curves were obtained on a Horiba JobinYvon Fluorolog-3 (Horiba, Kyoto, Japan) equipped with a pulsed 150 W xenon lamp using a time-correlated single-photon-counting (TCSPC) system. An Inductively Coupled Plasma Mass Spectroscopy (ICP-MS, Shimadzu 2030) from Shimadzu, Kyoto, Japan, was used for the actual compositions of NC analysis after NCs were digested under acid conditions. X-ray-diffraction (XRD) data were collected by a coupled theta: 2-theta scan on a Rigaku Ultima-III diffractometer equipped with Copper X-ray tube with Ni beta filter, parafocusing optics, computer-controlled slits, and D/Tex Ultra 1D strip detector.

The quantum yields (QYs) of the samples were determined using the equation:

QY_S_ = QY_R_ × (I_S_/I_R_) × (A_R_/A_S_) × (n_S_/n_R_)^2^

A is the absorbance, I is the integrated emission area, and n is the refractive index of the solvents for sample S and reference R.

Oxazine 170 (λ_em_ = 640 nm, QY = 63% in methanol) and Coumarin 153 (λ_em_ = 532 nm, QY = 54% in ethanol) were used as the standards for QY measurements. The same excitation wavelength was applied to both samples and quantum-yield standards during the QY measurements. The excitation wavelength was chosen in order to ensure a linear relationship between the concentration of the absorbing/emitting species and the intensity of emitted light (each sample was diluted to achieve an absorbance of ~0.05 at the exciting wavelength).

### 2.2. Synthesis of Host CuGaS-ZnS NCs and CuInS-ZnS NCs

In a typical synthesis of host CuGaS-ZnS NCs, CuI (0.0125 or 0.25 mmol), ODE (4 mL), DDT (0.5 mL), oleic acid (0.5 mL), and Ga(acac)_3_ (0.1 mmol) were added to a three-necked round-bottom flask (50 mL), which was equipped with a magnetic stir bar and a condenser. Under vacuum, the mixture was heated to 145 °C, and a clear solution was then obtained. Then, a flow of nitrogen was switched into the flask, the mixture was heated to 170 °C, and 0.6 mL of sulfur precursors (3 mL of oleylamine and 1 mL of DDT with 2 mmol sulfur) was swiftly injected into the flask. The mixture in the flask was further heated to 210 °C and remained at 210 °C for 40 min. The mixture was then heated to 240 °C for the alternative injection of the Zn precursor and sulfur precursor. For the injection of Zn and sulfur precursors, 0.6 mL of zinc precursors (2 mL of ODE, 1 mL of DDT, 1 mL of oleylamine, 2 mmol Zn(Ac)_2_) were added dropwise to the core NC solution. Subsequently, 0.6 mL of sulfur precursors were added dropwise. At 15 min intervals, the above addition process was repeated six times. Once the reaction was complete, the mixture was naturally cooled down. The host NCs were washed/purified using hexane/ethanol (or chloroform/acetone) and then dried under vacuum. Host CuInS-ZnS NCs were produced in the same way but using In(Ac)_3_.

### 2.3. Synthesis of Mn-Doped CuGaS-ZnS NCs and Mn-Doped CuInS-ZnS NCs

In a typical synthesis of Mn-doped CuGaS-ZnS NCs, ODE (4 mL), DDT (0.5 mL), oleic acid (0.5 mL), Ga(acac)_3_ (0.1 mmol), CuI (0.0125 or 0.25 mmol), and Mn(Ac)_2_ (0.003125–0.0375 mmol) were added to a three-necked round-bottom flask (50 mL), which was equipped with a magnetic stir bar and a condenser. The other steps are the same as for the host CuGaS-ZnS NCs. Mn-doped CuInS-ZnS NCs were produced in the same way but using In(Ac)_3_ instead of Ga(acac)_3_.

### 2.4. Decay Curve Fitting

The fluorescence-decay curves from the TCSPC measurements were measured under 405 nm excitation and fitted with a multi-exponential function I(t) = ∑A_i_exp (−t/τ_i_), where τ_i_ is the lifetime parameters, and A_i_ is the corresponding amplitude of τ_i_ at t = 0. The average lifetime for each sample was calculated as below.

τ_avg_ = ∑A_i_τ_i_^2^/∑A_i_τ_i_

### 2.5. Optical Setup for the Fluorescence Decay Testing on Mn-Doped NCs

The setup consisted of a 405 nm laser-excitation source (20 mW) and its circuit driver, a cuvette and its holder, a photosensor with a bandpass filter (607 ± 18 nm), and optical fibers for excitation-light delivery and emission-light collection [41]. The 405 nm laser diode was turned on/off through its circuit driver with repeated pulses at a 0.5 kHz rate with 40% duty cycle. The photosensor (photomultiplier tube, PMT) connected to a digital oscilloscope recorded the fluorescence characteristics of Mn-doped NCs under the pulsed 405 nm laser excitation.

## 3. Results and Discussion

With respect to technical approaches, our research went through the following steps. First, we prepared four types of I(II)-III-VI host NCs with four different peak emissions in the range of 500 nm to 570 nm, which are close to but less than the characteristic emission (i.e., 585 nm) from the ^4^T_1_ state to the ^6^A_1_ states of the Mn dopants. Because such materials follow the donor–acceptor model of light absorption and fluorescence emission and their fluorescence spectra have a full width at half maximum (FWHM) of around 100 nm, the peak-emission wavelengths at 500–570 nm will render these host NCs as excitable by visible light at 405 nm. These peak-emission wavelengths are less than the ideal Mn emission wavelength at 585 nm, and therefore the NC bandgaps are wide enough to incorporate Mn energy levels for Mn emission. Second, we doped the same amount of Mn dopants into each type of the host NCs through the homogenous-nucleation-doping approach (hot-injection-based synthesis) and explored how the different host NCs would affect the optical properties (i.e., the quantum yield (QY) and the fluorescence lifetime) of the doped NCs. Third, for each type of host NC, we changed the concentration of Mn (doped into the host NCs) over a wide range and investigated how the quantum yield, the fluorescence/absorption spectra, and the fluorescence lifetime of the doped NCs are affected by Mn concentrations. Through this study, the Mn-doped NCs, which can be further developed into time-gated probes, were achieved. Meanwhile, this study disclosed a generic synthetic approach with standard steps to produce optimal Mn-doped NCs for time-gated-probe development.

### 3.1. Effects of Host NC

**Figure 1:**
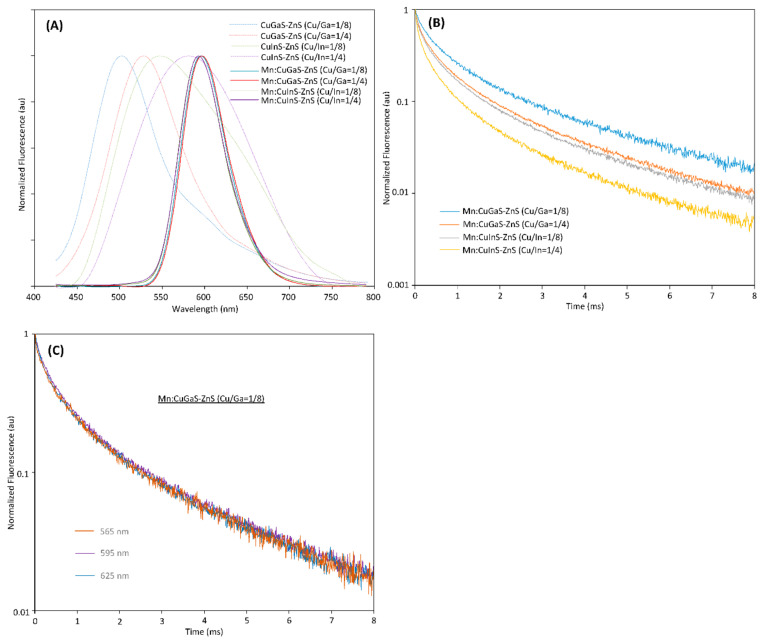
(**A**) Fluorescence spectra of four types of host NCs and four types of Mn-doped NCs (0.0125 mmol Mn-doped into each type of host NCs). (**B**) Fluorescence decays for four types of Mn-doped NCs (measured at 595 nm for all NC samples). (**C**) Fluorescence decays of Mn:CuGaS-ZnS (Cu/Ga = 1/8) NCs at 565, 595 and 625 nm.

From Figure 1A, it can be seen that the host NCs (without Mn doping) have peak emissions at around 500 nm, 530 nm, 545 nm and 570 nm, respectively. The QYs of all host NCs are low in the range of 0.5% to 5%, which indicate significant defects in all the prepared host NCs. As the Mn dopants are incorporated into each type of host NCs to form Mn-doped NCs, it can be seen that no matter which type of host NC is adopted, all of the Mn:CuGaS-ZnS and Mn:CuInS-ZnS NCs present almost the same emission spectra (Figure 1A). All the emission peaks are around 595 nm and all the emission spectra present almost the same shape profiles and the same FWHMs at around 60 nm. With respect to emission brightness, the QY is 47% for Mn:CuGaS-ZnS (Cu/Ga = 1/8) NCs, 12% for Mn:CuGaS-ZnS (Cu/Ga = 1/4) NCs, 14% for Mn:CuInS-ZnS (Cu/Ga = 1/8) NCs, and 3% for Mn:CuInS-ZnS (Cu/Ga = 1/4) NCs. Mn-doped NCs are significantly brighter than the corresponding host NCs. Figure S1 shows the absorption spectra for each pair of Mn-doped NCs and the corresponding host NCs. For host CuGaS-ZnS NCs with both Cu/Ga = 1/8 and Cu/Ga = 1/4, before and after Mn doping, their absorption spectra are almost similar. This indicates that Mn doping into these host NCs is not significantly affecting the bandgaps of the host NCs. The insets in Figure S1 show the fluorescence of Mn-doped NCs under 405 nm excitation. It is clear that the doped NCs are excitable by visible light (405 nm).

Figure 1B presents the fluorescence-decay curves of Mn-doped NCs; each curve corresponds to the Mn doping of each type of host NC. The decay of each type of Mn-doped NC was measured at their emission peak (595 nm). The decay-fitting analysis further indicates the average lifetime is 1.71 ms for Mn:CuGaS-ZnS (Cu/Ga = 1/8) NCs, 1.22 ms for Mn:CuGaS-ZnS (Cu/Ga = 1/4) NCs, 1.09 ms for Mn:CuInS-ZnS (Cu/Ga = 1/8) NCs, and 0.86 ms for Mn:CuInS-ZnS (Cu/Ga = 1/4) NCs. The emission lifetimes are listed in Table 1. For Mn:CuGaS-ZnS (Cu/Ga = 1/8) NCs, we also measured the decay curves at 565 nm and 625 nm, which are the lower and upper wavelengths at half-maximum of the NC fluorescence spectrum, respectively. Figure 1C shows the decays curves of this type of Mn-doped NC at 565 nm, 595 nm and 625 nm. All of these decay curves are almost overlapping and present very similar average lifetimes. Such a phenomenon is also observed for three other types of Mn-doped NCs (data not shown). The fluorescence-decay curves of four types of host NCs are presented in Supplementary Figure S2, which present lifetimes in the range of 300–600 ns as reported in the literature [22].

The shift in the emission spectrum and the lifetime before and after Mn doping clearly indicate the incorporation of Mn dopants into the host NCs, which further produces the Mn emission. The emission peak at ~595 nm is in the range of what was reported on the Mn emission in various host NCs [12,31], but close to the ideal Mn emission at ~585 nm. Mn-doped multinary NCs have been reported to produce Mn-related emission at 620 nm or up to 650 nm, and it is thought that the long emission is due to the significant coupling between the Mn d levels and the microenvironments of the host NCs [31]. Additionally, all four types of host NCs have a long emission tail (Figure 1A) indicating narrower energy gaps between the donor states and acceptor states for electron-hole recombination. But once Mn elements are doped into these types of host NCs, all types of Mn-doped NCs present almost the same emission spectral profiles. The possible reason is that the energy transfer to the Mn emission states is faster when compared to the energy transfer to the narrower energy gaps between the donor states and acceptor states, and thus Mn emission is dominant. Note that the use of Cu in the synthesis of Mn:CuGaS-ZnS (Cu/Ga = 1/4) NCs is double the amount of Cu used for Mn:CuGaS-ZnS (Cu/Ga = 1/8) NCs. From the recent literature [42], it is known that that in Cu-doped or Cu-based NCs, Cu introduces isolated and localized midgap d orbitals above the valence-band edge. In spite of the increase of the Cu amount in NC synthesis, the Cu midgap d orbitals may not stack up to be above the ^6^A_1_ states of Mn, and thus would not affect the Mn emission spectrum.

To confirm that the optical properties are associated with the composition ratios (Cu/Ga or Cu/In) and/or Mn dopants in NCs, the compositions of NCs were analyzed using ICP (Supplementary Table S1). In the synthesis of the host CuGaS-ZnS NCs, the Ga concentration was fixed at 0.1 mmol but the Cu concentration was doubled from 0.0125 to 0.025 mmol (Cu/Ga = 1/8→1/4). The ICP analyses of the NCs show that the actual Cu/Ga molar ratio is 0.12 for Cu/Ga = 1/8, and the one is 0.19 for Cu/Ga = 1/4. Although the actual molar ratios are slightly lower than the nominal ones, it is clear that the actual Cu/Ga molar ratio is doubled as the nominal Cu/Ga molar ratio is increased. The ICP analyses of the host CuInS-ZnS NCs show the similar conclusion. For Mn:CuGaS-ZnS or Mn:CuInS-ZnS NCs with Cu/Ga = 1/8 and 1/4 and Mn = 0.0125 mmol, the ICP analyses (Table S1) show that regardless of whether Ga or In is adopted in the NC synthesis, it can be seen that the actual molar ratio of Cu/Ga or Cu/In is increased as the nominal ratio of Cu/Ga or Cu/In is doubled, but the actual molar ratios of Mn/Ga or Mn/In are very close to each other when the actual/nominal molar ratio of Cu/Ga or Cu/In is doubled from 1/8 to 1/4. All Mn-doped NCs have almost overlapping emission spectra with a peak at ~595 nm (as shown in Figure 1A), and such overlapping spectra should be due to the doping of Mn into NCs instead of the molar-ratio change of Cu/Ga or Cu/In in NCs.

**Figure 2:**
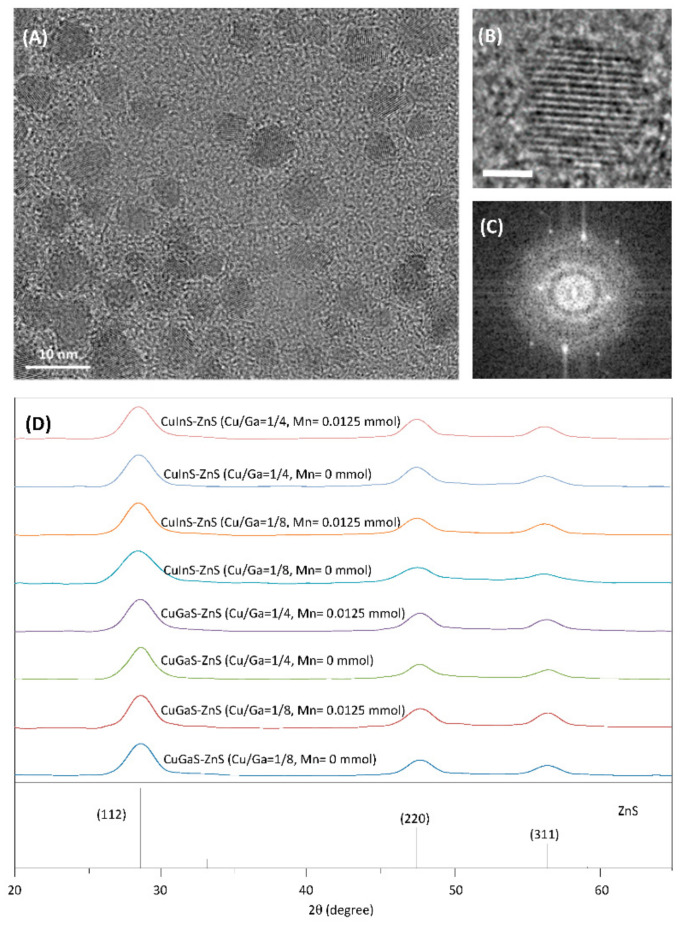
(**A**,**B**) TEM and high-resolution TEM (HRTEM) images of Mn:CuGaS-ZnS (Cu/Ga = 1/8, Mn = 0.0125 mmol) NCs. The scale bar in HRTEM image is 2 nm. (**C**) Fast-Fourier-Transform (FFT) pattern on the HRTEM image of the Mn:CuGaS-ZnS NCs. (**D**) XRD patterns of different types of host NCs and Mn-doped NCs (Mn = 0.0125 mmol for each type of host NCs).

Representative TEM and high-resolution TEM (HRTEM) images of Mn:CuGaS-ZnS (Cu/Ga = 1/8, Mn = 0.0125 mmol) NCs are shown in Figure 2A,B. The NC sizes are 5–7 nm. The Fast-Fourier-Transform (FFT) pattern of the HRTEM image is presented in Figure 2C. The interplanar distance in the lattice fringes as presented by HRTEM is found to be ~0.310 nm through analyzing the FFT pattern. This value is similar to the interplanar distance of the (111) plane in ZnS. The XRD patterns of four types of Mn-doped NCs and their corresponding host NCs are shown in Figure 2D; all samples present three apparent diffraction peaks at around 2θ = 28, 47, and 56°, which are similar to the (111), (220), and (311) planes of cubic ZnS (JCPDS #05-0566). No secondary phases such as MnS were identified. This indicates that different types of host NCs and Mn-doped NCs have similar NC crystal structures. It should be noted that in our synthesis, a slightly excessive amount of sulfur precursor was rapidly injected in order to wrap the metal precursors and form core NCs, and then Zn and sulfur precursors were alternatively injected dropwise to coat the core NCs. Ideally, the lattice of the core NCs should match with or be similar to that of the ZnS NCs for the ZnS shell growth. In this study, XRD measurement was applied to the core NCs to identify their crystal phases. Specifically, the XRD pattern of the CuGaS core NCs (Cu/Ga = 1/8) is shown in Figure S3, and the XRD pattern of CuGaS core NCs presents unique peaks that do not match with the CuS, Cu_2_S, Ga_2_S_3_, or CuGaS_2_ crystals. This XRD pattern is also different from that of the ZnS crystals, which is reasonable because the CuGaS core NCs under such stoichiometric deviations should not form a phase structure of the ZnS crystal. However, the XRD pattern of CuGaS-ZnS NCs (Cu/Ga = 1/8) has the major XRD characteristics of ZnS crystals. The reason for such a phase change during the NC synthesis is not clear and is still under investigation, but it is possible that once the Zn and sulfur precursors are coated onto the core NCs (with or without Mn doping), some Zn and sulfur elements could diffuse into and interact with the core NCs to restructure the core lattice and form a stable phase, and thus a newly reconstituted energy bandgap [35,43,44].

**Figure 3:**
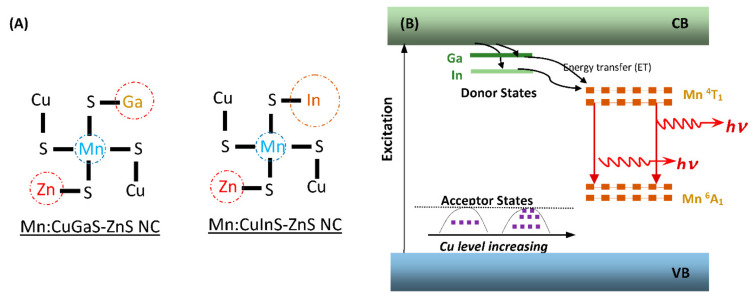
Illustration of possible fluorescence mechanisms of Mn-doped CuGaS-ZnS and CuInS-ZnS NCs. (**A**) Possible microenvironments surrounding Mn dopants (the coordinates of Mn with multiple ions in host NCs; the circle in illustration represents the relative atom size of Mn or Zn or Ga or In). (**B**) Possible energy-transfer (ET) pathways for Mn emission.

For all types of Mn-doped NCs, their fluorescence-decay curves were fitted with a multi-exponential model, and it was found that each curve best fits with a bi-exponential equation (0.97 < χ^2^< 1). As shown in Table 1, each type presents both a fast decay with a lifetime (τ1) in the range of 0.1–0.4 ms and a slow decay with a lifetime (τ_2_) in the range of 1–2.2 ms. The long lifetime (τ_2_) of these NCs should be attributed to Mn emission, considering the nature of Mn emission for long-fluorescence lifetimes, but the origin of the short lifetime (τ_1_) of these NCs is complex. In Table 1, it is observed that both the short lifetime and the long lifetime (thus the average lifetime) of Mn:CuGaS-ZnS NCs drops off when Cu/Ga ratio is increased from 1/8 to 1/4 (or the use of Cu in synthesis is double). An explanation is that when the Cu amount is increased in the NCs, the Mn dopants would have more Cu atoms in their surrounding environments and also be closer to the Cu atoms [45]. Thus, the interaction between Cu and Mn through their orbital electron clouds is stronger. Additionally, more Cu atoms in NCs could induce more NC lattice strains/defects, and thus the interaction between Cu-induced lattice strains/defects and Mn would be stronger. Such enhanced interactions could further strengthen the splitting of the Mn ^4^T_1_ and ^6^A_1_ states or cause the reconstitution of the Mn emission states for a faster decay. Note that the interaction would cause the further decrease of the lifetime (both the short lifetime and the long lifetime). The long lifetime is thought be due to the relatively isolated Mn dopants in the NCs [31,46,47]. The number of relatively isolated Mn dopants could be reduced with the incorporation of more Cu atoms into the host NCs, which would result in the decrease in the long lifetime. Table 1 also shows that both the short lifetime and the long lifetime of Mn:CuInS-ZnS (Cu/In = 1/8) NCs is less than that of Mn:CuGaS-ZnS (Cu/Ga = 1/8) NCs when indium instead of gallium is used for the host NC synthesis. Compared to gallium, indium is much larger in size (including its electron clouds and nucleus), and its interaction with the Mn dopants (reflected by the fluorescence peak wavelength and lifetime of Mn emission) would be stronger due to the shorter distance between In and Mn. Such an interaction could cause the decrease in both the short lifetime and the long lifetime due to the same logistics as the explanation of more Cu atoms for a shorter average lifetime. From Table 1, it can be seen that Mn:CuInS-ZnS (Cu/In = 1/4) NCs have the lowest average lifetime, which should be due to the synergistic efforts of more Cu atoms and the larger size of indium in NCs. On the basis of the above discussion, Figure 3A illustrates the possible microenvironments surrounding Mn dopants in Mn-doped CuGaS-ZnS and CuInS-ZnS NCs. This illustration reflects the size effect of Ga and In on their interaction strength with Mn through their electron clouds. It also depicts more Cu atoms surrounding each Mn dopant to induce stronger interaction with the Mn dopant if the Cu amount is increased in the NC synthesis. Figure 3B illustrates the possible electronic energy structure of Mn-doped NCs for Mn emission. In the donor–acceptor model of these NCs, Ga and In contribute to the donor states and Cu (in different levels) contributes to the acceptor states. In spite of the double amount of Cu in the synthesis, the Cu midgap d orbitals may still be at low levels [41] and may not stack up to be above the ^6^A^1^ states of Mn to affect the Mn emission. As illustrated in Figure 3B, upon photoexcitation, excitation energy can be transferred from the conduction band to donors and then to the ^4^T_1_ states of Mn dopants for Mn emission, and the microenvironment surrounding the Mn dopants will further determine the lifetime of Mn emission.

### 3.2. Effects of Mn Concentration in NCs

With respect to brightness (QY), lifetime, and low energy for excitation (excitable by visible light at 405 nm), as indicated in Table 1, Mn:CuGaS-ZnS (Cu/Ga = 1/8) NCs are optimal and have potential to be further developed as time-gated probes for TGFM, and thus the effects of Mn concentration on this type of host NC were further investigated.

**Figure 4:**
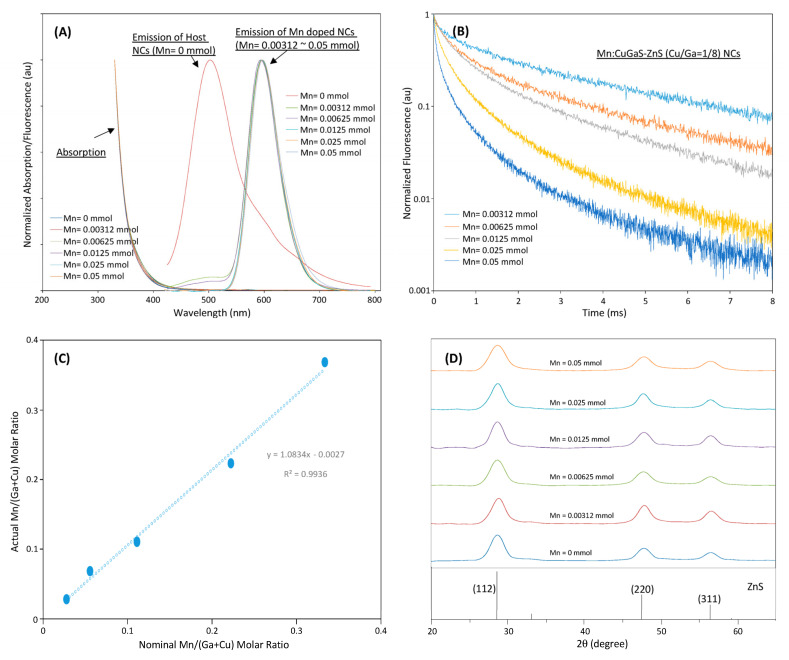
(**A**) Fluorescence and absorption spectra of CuGaS-ZnS (Cu/Ga = 1/8) NCs and Mn:CuGaS-ZnS (Cu/Ga = 1/8) NCs with a wide Mn-concentration range in synthesis. (**B**) Fluorescence decays of Mn:CuGaS-ZnS (Cu/Ga = 1/8) NCs at their peak wavelengths with different Mn concentrations in synthesis. (**C**) ICP-based actual Mn/(Ga + Cu) molar ratios of Mn:CuGaS-ZnS (Cu/Ga =1/8) NCs with a series of Mn concentrations in synthesis. (**D**) XRD patterns of Mn:CuGaS-ZnS (Cu/Ga = 1/8, Mn = 0.0125 mmol) NCs with different Mn concentrations in synthesis.

Figure 4A shows the emission-absorption spectra of Mn:CuGaS-ZnS (Cu/Ga = 1/8) NCs with different Mn concentrations in synthesis. The emission spectra of all Mn-doped NCs present almost the same emission spectra. All emission peaks are around 595 nm except that of Mn = 0.05 mmol at around 600 nm. All emission spectra present almost the same shape profiles and the same FWHMs at around 60 nm. For the emission spectra of the NCs that used Mn = 0.00312 and 0.00625 mmol in their synthesis, a tail is observed in the range of 450–510 nm. It is believed that due to the limited Mn dopants in the synthesis, not all the donor–acceptor emissions are quenched, and a small amount of light is still from the donor–acceptor emissions [37,38,39].

Figure 4B shows the fluorescence-decay curves of Mn-doped NCs. Each curve corresponds to a distinct Mn concentration in the synthesis. The decay of each type of Mn-doped NC was measured at their emission peak. Figure 4B as well as the further decay-fitting analysis indicates that the longest average lifetime at 3.67 ms is achieved when the lowest Mn concentration is used in the synthesis. As more and more Mn dopants are incorporated into NCs in the synthesis, both the short lifetime and the long lifetime (thus the average lifetime) keep dropping. It should be noted that for each type of Mn-doped NC, we measured the decay curves at the lower and upper wavelengths at the half maximum of the NC fluorescence spectrum, respectively. All of these decay curves are almost overlapping with the curve measured at their peak wavelength, presenting very similar average lifetimes to each other. Specifically, we measured the fluorescence decays at 500 nm for the conditions of Mn = 0.00321 mmol and 0.00625 mmol, and fast decays (or short lifetimes at several hundreds of nanoseconds) were observed (data not shown). These measurements confirm that the tails result from the donor–acceptor emissions. The emission lifetimes (measurement at the fluorescence peaks of all investigated NCs) as well as their QYs are listed in Table 2. From Table 2, it can be seen that Mn:CuGaS-ZnS (Cu/Ga = 1/8) NCs have the highest QY (47%), and the QYs of all other samples are still good (>10%). The overall range of the average lifetime spans from 0.27 ms to 3.67 ms. The wide range of the average lifetime may benefit multiplexing biosensing/imaging. Some NCs (Mn = 0.00312 mmol) with a QY of 19% and a lifetime of 3.67 ms should be suitable to develop camera-based, low-cost, and portable bio-detection systems.

In the synthesis of all Mn:CuGaS-ZnS (Cu/Ga = 1/8) NCs, the synthesis concentrations of Cu and Ga remained unchanged at 0.0125 mmol and 0.1 mmol, respectively, but the Mn concentration in the synthesis was widely changed from 0.00312 to 0.05 mmol. The ICP analyses (Supplementary Table S2) show that in all the NCs that were doped with different Mn concentrations, the Cu and Ga molar concentrations are kept almost unchanged, but the Mn molar concentration is observed to increase with the increase in Mn concentration in the synthesis. Figure 4C shows the actual Mn/(Ga + Cu) molar ratio versus the nominal Mn/(Ga + Cu) molar ratio; they are very close to each other, but the actual molar ratio is slightly higher than the corresponding nominal molar ratio. The ICP analyses clearly indicate that the optical properties (emission spectra and lifetimes) should be associated with the concentration of Mn in the NCs.

Figure 4D shows the XRD patterns of Mn:CuGaS NCs (Cu/Ga = 1/8) under the different Mn concentrations. As indicated in this figure, all the samples clearly present three apparent diffraction peaks at around 2θ = 28, 47, and 56°, and can be explained in the same way as in Figure 2D. No secondary phases such as MnS were observable even for the samples synthesized with a higher Mn concentration. This suggests that the different Mn concentrations do not significantly change the NC crystal structure.

With respect to the NC optical properties, Mn in the host NCs could interact with the host lattice to aggravate or lessen lattice strains or defects depending on the Mn concentration [13,14,15,16,48,49,50,51,52,53,54,55]. This could be the reason for the highest QY of NCs being for Mn = 0.0125 mmol, but there were lower QYs of other NCs with more or less Mn concentrations. The lifetime dependence on the Mn concentration may be explained through Mn–Mn interactions [47,48,49,50,51]. When more Mn dopants are incorporated into host NCs, Mn dopants become clustered and the Mn–Mn distance is shorter. The shorter Mn–Mn distance will strengthen the Mn–Mn spin coupling or exchange the interaction of coupled Mn pairs, thereby partially lifting the spin-selection rule (Figure 3B). As a result, Mn emission has a shorter lifetime as the Mn concentration increases. It should be noted that as the Mn concentration increases to a very high level (e.g., 0.025 or 0.05 mmol), more lattice strains/defects could be formed, contributing to the fast emission decay.

In this study, we also doped Mn at different concentrations (0.003125, 0.00625 and 0.0125 mmol) into other three types of host NCs to investigate their optical properties. Supplementary Tables S3–S5 present the optical parameters. Through comparing the parameters in these tables or comparing with other previous work [45], it can be seen that the Mn:CuGaS-ZnS (Cu/Ga = 1/8) NCs have better optical qualities (higher QYs and/or longer lifetimes). So, it could be concluded that the choice of host NC and Mn concentration do is critical to produce Mn-doped NCs with high-quality optical properties.

### 3.3. Demonstration of Time-Domain Fluorescence Characteristics under Pulsed 405 nm Laser Excitation and Bandpass-Filter-Based Emission Collection

**Figure 5:**
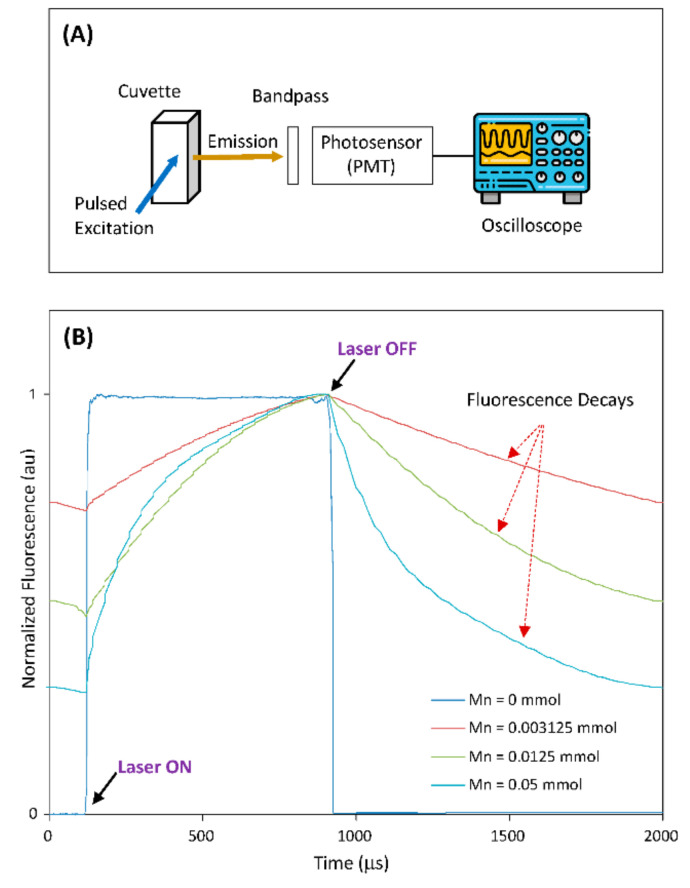
(**A**) The setup illustration of the optical-measurement system. (**B**) The normalized system responses from all selected NCs within a period of laser pulse (host CuGaS-ZnS with Cu/Ga = 1/8, and Mn-doped CuGaS-ZnS with Cu/Ga = 1/8 and Mn = 0.003215, 0.0125, 0.05 mmol, respectively).

In most applications of TGFM, probes with a long fluorescence lifetime are often excited using a light source (e.g., laser diode) and their fluorescence signals are collected by photosensor (such as PMT) through a bandpass filter. To demonstrate the potential of the Mn-doped NCs as probes for practical TGFM applications, we built an optical-measurement system to measure the time-domain fluorescence characteristics of Mn-doped NCs, as illustrated in Figure 5A. In this demonstration, the host CuGaS-ZnS (Cu/Ga = 1/8) NCs and Mn-doped CuGaS-ZnS (Cu/Ga = 1/8, Mn = 0.003215, 0.0125, 0.05 mmol) NCs were selected. Figure 5B shows the normalized system responses from all the selected NCs (suspended in chloroform) within a period of laser pulse. It is clear that after the laser turns off, the fluorescence signal of the host NCs without Mn doping drops off very fast (due to their lifetime of several hundreds of nanoseconds), but other Mn-doped NCs present long fluorescence decays (due to lifetimes at around 1 ms or longer). As Mn concentration is increased from 0.003125 to 0.0125 to 0.05 mmol, the Mn-doped NCs present the slowest decay to the fastest decay, which matches the change trend of the measured lifetime vs. the change in Mn concentration (as shown in Table 2). This demonstration clearly indicates that the fluorescence decay of Mn-doped NCs would make it feasible for time-gated instruments to capture fluorescence signals after the laser is off [41]. On the other hand, since NC-surface-modification approaches for phase transfer and bioconjugation are well-established [56], these surface-modification approaches are readily applicable to these Mn-doped NCs for broad time-gated biosensing/imaging applications. Lastly, it should be pointed out that our work showed an approach to produce NCs with different lifetimes but good brightness as indicated in Table 1 and Table 2 as well as Tables S3–S5. Appropriate NCs could be further used in multiplexing and time-gated fluorescence measurements in biosensing/imaging.

## 4. Conclusions

In summary, four types of host CuGa(In)S-ZnS NCs of green emission colors (peak wavelengths at 500–570 nm) were prepared, and each type of host NC was doped with Mn at different concentrations in the synthesis of Mn-doped NCs. It was found that the optical properties (brightness, fluorescence/absorption spectra, and fluorescence lifetime) of Mn-doped NCs are significantly affected by different host NCs and Mn concentrations. With optimal synthetic conditions, a library of Mn-doped NCs was achieved that possessed high brightness, low-energy (405 nm visible light) excitation, and long lifetimes up to milliseconds. Additionally, the time-domain fluorescence characteristics of some optimal Mn-doped NCs were measured under pulsed 405 nm laser excitation and bandpass-filter-based emission collection. The measurement results indicate that these optimal Mn-doped NCs present a long fluorescence decay under excitation at 405 nm, which makes them feasible in TGFM-based biosensing/imaging.

## Figures and Tables

**Table 1:** Effect of host NC on optical properties of Mn-doped NCs.

	Composition Ratio (mmol)	Cu/Ga = 1/8	Cu/Ga = 1/4	Cu/In = 1/8	Cu/In = 1/4
Without Mn Doping	Wavelength (nm)	~500	~530	~545	~570
	QY	2%	1%	5%	0.5%
	τ_1_ (ns)	29	20	71	88
	A_1_	91.2%	98.7%	86.5%	80.6%
	τ_2_ (ns)	548	518	840	747
	A_2_	8.8%	1.3%	13.5%	19.4%
	Avg τ (ns)	365	371	569	530
With Mn Doping (Mn = 0.0125 mmol)	Wavelength (nm)	~595	~595	~595	~595
	QY	47%	12%	14%	3%
	τ_1_ (ms)	0.34	0.22	0.19	0.11
	A_1_	70.0%	75.2%	76.6%	68.2%
	τ_2_ (ms)	2.20	1.63	1.47	1.03
	A_2_	30.0%	24.8%	23.4%	31.8%
	Avg τ (ms)	1.71	1.22	1.09	0.86

**Table 2:** Effects of Mn concentration on optical properties of Mn:CuGaS-ZnS (Cu/Ga = 1/8) NCs.

Mn Conc. in Synthesis (mmol)	Wavelength (nm)	QY	τ_1_ (ms)	A_1_	τ_2_ (ms)	A_2_	Avg τ (ms)
0.003125	~595	18.8%	0.51	51.6%	4.09	48.4%	3.67
0.00625	~595	16.5%	0.39	70.0%	2.99	30.0%	2.38
0.0125	~595	47.4%	0.34	69.9%	2.20	30.1%	1.71
0.025	~595	28.7%	0.14	78.0%	1.02	36.0%	0.73
0.05	~600	11.7%	0.06	94.9%	0.64	5.1%	0.27

## Data Availability

The data presented in this study are available in this article (and Supplementary Materials).

## Supplementary Materials

The following supporting information can be downloaded at: www.mdpi.com/article/10.3390/nano12060994/s1, Figure S1: Absorption spectra of each type of host NCs and its corresponding Mn-doped NCs. The inset images show the fluorescence of all types of Mn-doped NCs under the exposure of a 405 nm laser beam, indicating all Mn-doped NCs are excitable at 405 nm; Figure S2: Fluorescence decays of four types of host NCs (measured at their fluorescence peak wavelengths); Figure S3: XRD pattern of CuGaS NCs (Cu/Ga = 1/8); Table S1: ICP analysis data for host NCs and the corresponding Mn-doped NCs; Table S2: ICP analysis data for Mn-doped NCs with different Mn concentrations in synthesis; Table S3: Effects of Mn concentration on optical properties of Mn:CuGaS-ZnS (Cu/Ga = 1/4) NCs; Table S4: Effects of Mn concentration on optical properties of Mn:CuInS-ZnS (Cu/In = 1/8) NCs; Table S5: Effect of Mn concentration on optical properties of Mn:CuInS-ZnS (Cu/In = 1/4) NCs.
