# Supplementary material for: Optical Properties of Mn-Doped CuGa(In)S-ZnS Nanocrystals (NCs): Effects of Host NC and Mn Concentration
Source: Nanomaterials (Basel). 2022 Mar 17;12(6):994. doi: 10.3390/nano12060994 (PMC8956066; doi:10.3390/nano12060994)
Supplement: Supplementary file 1 [file nanomaterials-12-00994-s001.zip › nanomaterials-1637210-supplementary.pdf]

nanomaterials

Supplementary Material

Optical Properties of Mn-Doped CuGa(In)S-ZnS Nanocrystals (NCs): Effects of Host NC and Mn Concentration

Bryan Lee [1,2], Tristan Hegseth [1], and Xiaoshan Zhu [1,2,*]

[1] Department of Electrical and Biomedical Engineering, University of Nevada Reno, Reno, NV 89557, USA; bryanlee@nevada.unr.edu (B.L.); tristanhegseth@nevada.unr.edu (T.H.)
[2] Biomedical Engineering Program, University of Nevada Reno, Reno, NV 89557, USA
* Correspondence: xzhu@unr.edu

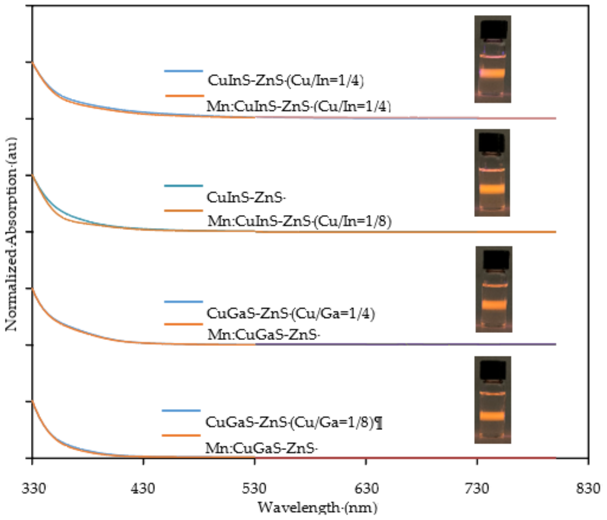

Figure S1. Absorption spectra of each type of host NCs and its corresponding Mn doped NCs (0.0125 mmol Mn doped into each type of host NCs). The inset images show the fluorescence of all types of Mn doped NCs under the exposure of a 405 nm laser beam, indicating all Mn doped NCs are excitable at 405 nm.

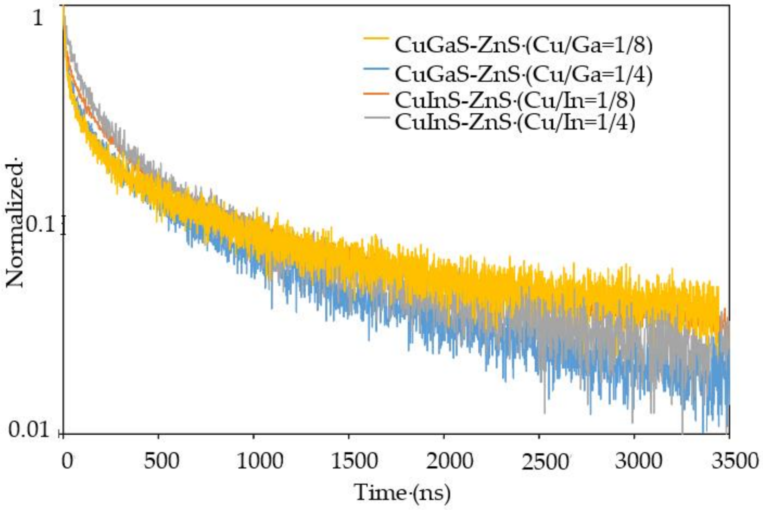

Figure S2. Fluorescence decays of four types of host NCs (measured at their fluorescence peak wavelengths).

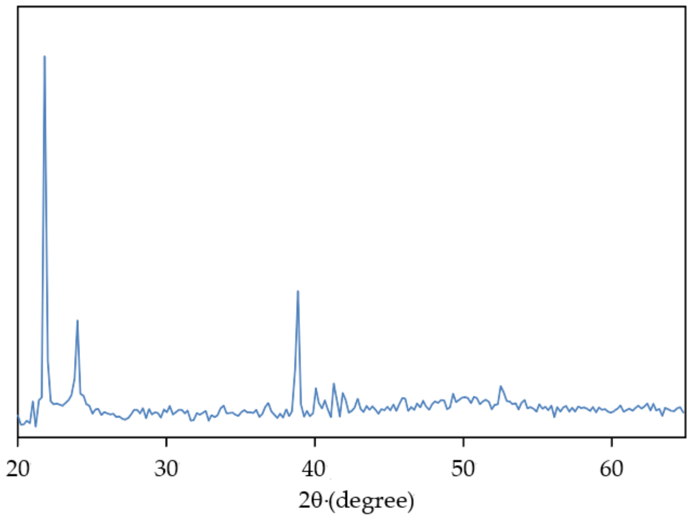

Figure S3. XRD pattern of CuGaS NCs (Cu/Ga = 1/8).

Table S1. ICP analysis data for host NCs and the corresponding Mn doped NCs.

	Nominal molar ratios				Actual milli-mole of element per 1 g NCs					Actual molar ratios			
	**Cu/Ga**	**Cu/In**	**Mn/Ga**	**Mn/In**	**Cu**	**Ga**	**In**	**Mn**	**Zn**	**Cu/Ga**	**Cu/In**	**Mn/Ga**	**Mn/In**
CuGaS-ZnS	1/8	NA	0	NA	0.075	0.608	0.000	0.000	11.698	0.123	NA	0.000	NA
	1/4	NA	0	NA	0.134	0.691	0.000	0.000	10.198	0.195	NA	0.000	NA
CuInS-ZnS	NA	1/8	NA	0	0.091	0.000	0.887	0.000	8.602	NA	0.102	NA	0.000
	NA	1/4	NA	0	0.154	0.000	0.875	0.000	8.021	NA	0.176	NA	0.000
Mn:CuGaS-ZnS	1/8	NA	1/8	NA	0.075	0.558	0.000	0.070	9.612	0.134	NA	0.125	NA
	1/4	NA	1/8	NA	0.160	0.661	0.000	0.098	11.380	0.243	NA	0.148	NA
Mn:CuInS-ZnS	NA	1/8	NA	1/8	0.076	0.000	0.720	0.094	10.864	NA	0.106	NA	0.130
	NA	1/4	NA	1/8	0.138	0.001	0.749	0.079	10.407	NA	0.184	NA	0.106

Table S2. ICP analysis data for Mn doped NCs with different Mn concentrations in synthesis.

	**Mn Conc. (mmol)**	**Nominal Mn/(Ga + Cu)**	**Actual milli-mole of element per 1 g NCs**					**Actual Mn/(Ga + Cu)**
			**Cu**	**Ga**	**In**	**Mn**	**Zn**	
**Mn:CuGaS-ZnS**	0.003125	0.028	0.068	0.635	0.000	0.020	11.011	0.028
	0.00625	0.056	0.079	0.647	0.000	0.050	11.233	0.068
	0.0125	0.111	0.075	0.558	0.000	0.070	9.612	0.111
	0.025	0.222	0.061	0.585	0.000	0.144	11.080	0.223
	0.0375	0.333	0.063	0.556	0.000	0.228	10.530	0.369

Table S3. Effects of Mn concentration on optical properties of Mn:CuGaS-ZnS (Cu/Ga = 1/4) NCs.

**Mn Conc. in Synthesis (mmol)**	**Wavelength (nm)**	**QY**	**τ_1 (ms)**	**A_1**	**τ_2 (ms)**	**A_2**	**Avg τ (ms)**
0.003125	~ 595	4.8%	0.28	69.8%	3.07	30.2%	2.58
0.00625	~ 595	12.4%	0.28	73.7%	2.50	26.3%	1.97
0.0125	~ 595	11.7%	0.22	75.2%	1.63	24.8%	1.22

Table S4. Effects of Mn concentration on optical properties of Mn:CuInS-ZnS (Cu/In = 1/8) NCs.

**Mn Conc. in Synthesis (mmol)**	**Wavelength (nm)**	**QY**	**τ_1 (ms)**	**A_1**	**τ_2 (ms)**	**A_2**	**Avg τ (ms)**

0.003125	~ 595	5.3%	0.31	65.6%	3.03	34.4%	2.59
0.00625	~ 595	14.7%	0.32	69.5%	2.4	30.5%	1.93
0.0125	~ 595	13.7%	0.19	76.6%	1.47	23.4%	1.09

Table S5. Effect of Mn concentration on optical properties of Mn:CuInS-ZnS (Cu/In = 1/4) NCs.

Mn Conc. in Synthesis (mmol)	Wavelength (nm)	QY	τ_1 (ms)	A_1	τ_2 (ms)	A_2	Avg τ (ms)
0.003125	~ 595	0.8%	0.21	79.1%	2.21	20.9%	1.68
0.00625	~ 595	1.9%	0.21	78.7%	1.89	21.3%	1.39
0.0125	~ 595	2.7%	0.11	68.2%	1.02	31.8%	0.86
